# Galectin-3 Facilitates Cell Motility in Gastric Cancer by Up-Regulating Protease-Activated Receptor-1(PAR-1) and Matrix Metalloproteinase-1(MMP-1)

**DOI:** 10.1371/journal.pone.0025103

**Published:** 2011-09-22

**Authors:** Seok-Jun Kim, Ji-Young Shin, Kang-Duck Lee, Young-Ki Bae, Il-Ju Choi, Seok Hee Park, Kyung-Hee Chun

**Affiliations:** 1 Gastric Cancer Branch, Division of Translational and Clinical Research I, National Cancer Center Research Institute and Hospital, Jungbalsan-ro, Ilsandong-gu, Goyang-si, Gyeonggi-do, Republic of Korea; 2 Cancer Experimental Recourses Branch, Division of Cancer Biology, National Cancer Center, Ilsandong-gu, Goyang-si, Gyeonggi-do, Republic of Korea; 3 Department of Biological Science, Sungkyunkwan University, Jangan-gu, Suwon-si, Gyeonggi-do, Republic of Korea; Institut de Génomique Fonctionnelle de Lyon, France

## Abstract

**Background:**

Galectin-3 is known to regulate cancer metastasis. However, the underlying mechanism has not been defined. Through the DNA microarray studies after galectin-3 silencing, we demonstrated here that galectin-3 plays a key role in up-regulating the expressions of protease-activated receptor-1(PAR-1) and matrix metalloproteinase-1(MMP-1) PAR-1 thereby promoting gastric cancer metastasis.

**Methodology/Principal Findings:**

We examined the expression levels of Galectin-3, PAR-1, and MMP-1 in gastric cancer patient tissues and also the effects of silencing these proteins with specific siRNAs and of over-expressing them using specific lenti-viral constructs. We also employed zebrafish embryo model for analysis of *in vivo* gastric cancer cell invasion. These studies demonstrated that: a) galectin-3 silencing decreases the expression of PAR-1. b) galectin-3 over-expression increases cell migration and invasion and this increase can be reversed by PAR-1 silencing, indicating that galectin-3 increases cell migration and invasion via PAR-1 up-regulation. c) galectin-3 directly interacts with AP-1 transcriptional factor, and this complex binds to PAR-1 promoter and drives PAR-1 transcription. d) galectin-3 also amplifies phospho-paxillin, a PAR-1 downstream target, by increasing MMP-1 expression. MMP-1 silencing blocks phospho-paxillin amplification and cell invasion caused by galectin-3 over-expression. e) Silencing of either galectin-3, PAR-1 or MMP-1 significantly reduced cell migration into the vessels in zebrafish embryo model. f) Galectin-3, PAR-1, and MMP-1 are highly expressed and co-localized in malignant tissues from gastric cancer patients.

**Conclusions/Significance:**

Galectin-3 plays the key role of activating cell surface receptor through production of protease and boosts gastric cancer metastasis. Galectin-3 has the potential to serve as a useful pharmacological target for prevention of gastric cancer metastasis.

## Introduction

Cancers that spread (metastasize) from their original site to another area of the body, are called metastatic cancers. metastatic cancers have poor prognosis and high mortality [1]. A full understanding of the biological mechanism(s) involved in metastasis and rational approaches to preventing or controlling metastasis can lead to practical approaches for improving survival rate of patients afflicted with metastatic cancers. In previous studies, we found that galectin-3 increases gastric cancer cell motility by up-regulating fascin-1, an actin-bundling cytoskeletal protein [2]. Galectin-3 is a 31 kDa carbohydrate-recognition protein, involved in tumorigenesis, cancer cell growth and metastasis [3], [4], [5] and its high expression in several human cancers has been found to correlate with the poor prognosis of metastatic cancers.

To clarify the role of galectin-3 in cancer metastasis, we knocked-down its expression in gastric cancer cells using its siRNA and examined the results in gene expression by DNA microarray analysis [6]. Among previous results, we found significant reduction in the level of protease-activated receptor-1 (PAR-1), a member of the family of transmembrane G-protein-coupled receptors [7], and its activator, matrix metalloprotease-1(MMP-1) (Figure S1). Cleaved PAR-1 is auto-phosphorylated and transduced by extracellular signal(s) through various pathways, and plays a critical role in tumor metastasis [7], [8], [9]. Over-expression of PAR-1 has been reported in several cancers, including melanomas, breast and gastric cancers. MMP-1 is also up-regulated in a wide variety of advanced cancers, and a significant negative correlation has been observed between its expression and patient survival [10], [11], [12], [13]. Also several studies found that MMP-1 plays a crucial role in metastasis of breast cancer [14], liver and colon cancers [15], and gastric cancers [16], [17], among others. It appears that once secreted, MMP-1 promotes cancer cell invasion through degradation of extracellular matrices and/or submucosal layers of lymphatics [10], [14]. Numerous studies demonstrated that inhibition of MMPs leads to the inhibition of cell invasion [18], [19].

hese findings led us to hypothesize that galectin-3, PAR-1 and MMP-1 may be involved in enhancing the migration and invasion of gastric cancer. To test this hypothesis, we conducted studies for their role in gastric cancer cells. In both *in vitro* and *in vivo* studies, we used zebrafish embryo model for determining their effects on migration and invasion of cancer cells with living organelles. We also determined the expression levels of galectin-3, PAR-1 and MMP-1 in malignant tissues from gastric cancer patients for clinical correlations between these proteins in human specimens.

## Results

### Silencing galectin-3 or PAR-1 reduces the migration and invasion of human gastric cancer cells

**Figure 1:**
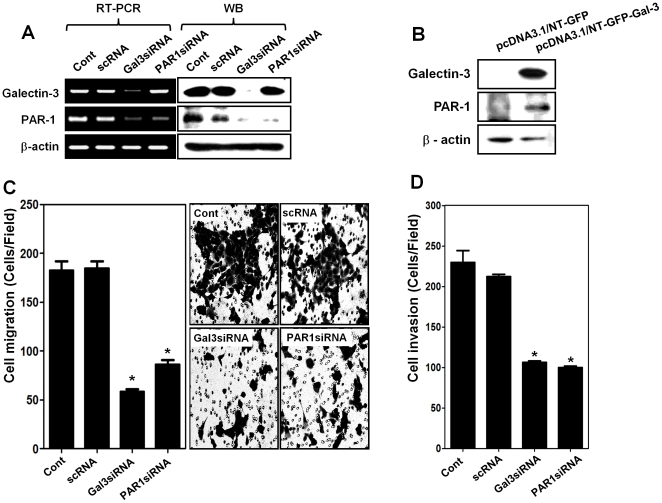
Knock down studies showing that galectin-3 regulates PAR-1 expression and gastric cancer cell migration and invasion. **A,** mRNA and protein expression levels after transfection of human gastric cancer MKN-28 cells with 20 nM scRNA, or siRNAs of galectin-3 or PAR-1. Total RNA and protein obtained after transfection for 48 hr. Cells were harvested and analyzed by RT-PCR and western blotting. β-actin was used as a loading control. **B,** Protein levels of galectin-3 and PAR-1 by western blotting after transfection with pcDNA3.1/NT-GFP-Galectin-3 and vector control of pcDNA3.1/NT-GFP in SNU-638 cells. **C,** Cell migration assay performed of galectin-3 or PAR-1 in silenced MKN-28 cells. The results were present as a histogram (* *p*<0.001 vs. Cont group), and cell photos of cell migration assays. **D,** Histogram was present that cell invasion assays of galectin-3 or PAR-1 in silenced MKN-28 cells (* *p*<0.001 vs. Cont group).

The expression levels of galectin-3 and PAR-1 were high in most gastric cancer cell lines. We silenced galectin-3 and PAR-1 in MKN-28 cells employing the respective siRNAs. Treatment with galectin-3 siRNA decreased expression levels of both galectin-3 and PAR-1, whereas PAR-1 siRNA treatment decreased only PAR-1 expression while no difference was observed in galectin-3 (Figure 1A). Transfection of SNU-638 cells, which originally display no expression galectin-3, with galectin-3-encoding plasmid resulted in increased expression of both PAR-1 and galectin-3 (Figure 1B). Silencing either galectin-3 or PAR-1 reduced total numbers of migrating (*p*<0.001) (Figure 1C) and invading cells (*p*<0.001) (Figure 1D), almost by half. These data showed that galectin-3 increased PAR-1 expression and both galectin-3 and PAR-1 modulated gastric cancer cell migration and invasion.

### Galectin-3 enhances gastric cancer cell migration/invasion by increasing PAR-1 expression

**Figure 2:**
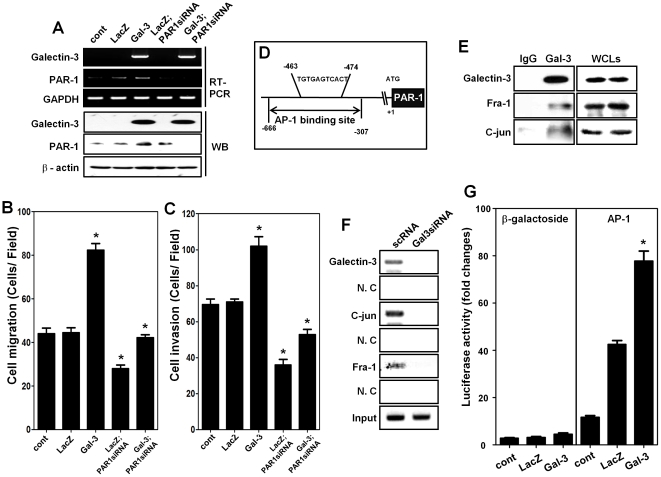
Galectin-3 enhances gastric cancer cell migration/invasion by increasing PAR-1 expression. **A,** mRNA and protein levels of galectin-3 and PAR-1 detected by RT-PCR and western blot analysis in SNU-638 cells, infected with lenti-virus containing LacZ or galectin-3, and then transfected with PAR-1 siRNA or scRNA for a negative control. β-actin was used as a loading control. **B** and **C**, Migration (**B**) and invasion (**C**) assays were conducted of SNU-638 cells infected with lenti-virus containing LacZ or galectin-3 cells, and then transfected with PAR-1 siRNA or scRNA for a negative control. Results was shown as histogram (* *p*<0.001 vs. Cont group). **D,** Schematic model of PAR-1 promoter with AP-1 binding site, Primers used for ChIP assay were prepared to detect AP-1 binding site (−463∼−474) from −666 to −307. **E,** Galectin-3 interacts with c-Jun and fra-1 (as, an AP-1 complex [20]) in MKN-28 cells. Immunoprecipitation was performed as described in “Material and Methods”, and then galectin-3, c-Jun and fra-1 detected by western blot analysis. Whole cell lysates (WCLs) were used as a positive control. **F,** Analysis of chromatin immunoprecipitation assay using antibodies to galectin-3, c-Jun and Fra-1 in MKN-28 cells transfected with scRNA and galectin-3 siRNA. PCR primer for the PAR-1 gene promoter was used to detect promoter fragment in immunoprecipitates. Input lane, total genomic DNA used as control for the PCR reaction. **G,** Luciferase activity of AP-1 in lacZ and galectin-3 over-expressing cells. Luciferase assay was performed using AP-1 expression luciferase vector transfection to lacZ and galectin-3 over-expressing cells (* *p*<0.001 vs. Cont). β-galactoside was used as negative control.

We examined the relationship between galectin-3-induced increases in cell migration and invasion on the one hand and PAR-1 expression on the other hand. We infected SNU638 cells with a lentivirus containing the galectin-3 expression cassette, and examined the expressions of galectin-3 and PAR-1, and measured cell migration. We found that over-expression of galectin-3 was accompanied by increased PAR-1 mRNA and protein expressions (Figure 2A), as well as cell migration (*p*<0.001) (Figure 2B) and cell invasion (*p*<0.001) activities (Figure 2C). These increases were reduced by PAR-1 silencing. These results suggested that galectin-3 promoted the migration and invasion of gastric cancer cells through up-regulation of PAR-1.

### Galectin-3 promotes PAR-1 transcription through interaction with AP-1 complex

We then attempted to elucidate how galectin-3 regulates PAR-1 expression. Because it was reported that AP-1 transcriptional activity is regulated by galectin-3 [20], we focused on the role of this transcription factor in this regard (Figure 2D). We performed immunoprecipitation studies and determined that galectin-3 directly interacted with both Fra-1 and c-Jun in the AP-1 complex, and that this interaction was associated with up-regulation of AP-1 transcriptional activity (Figure 2E). Next, we examined the DNA binding activity of AP-1 with and without galectin-3 by ChIP assays (Figure 2F). We found that AP-1 complex bound the promoter of PAR-1 in the presence of galectin-3, but not in its absence. We also assessed the transcriptional activity of AP-1 by luciferase assay after transfection of a reporter plasmid containing AP-1 binding sequence in front of a luciferase-driving minimal promoter in LacZ or galectin-3 over-expressing SNU-638 cells (*p*<0.001) (Figure 2G). The transcriptional activity of AP-1 significantly increased in galectin-3 over-expressing cells, but not in LacZ over-expressing cells. These results suggested that galectin-3 facilitated the binding of the PAR-1 promoter by interacting with AP-1 complex, thereby increasing PAR-1 expression by transcriptional regulation.

### Galectin-3 increases the expression of MMP-1 and the activation of PAR-1 signaling

**Figure 3:**
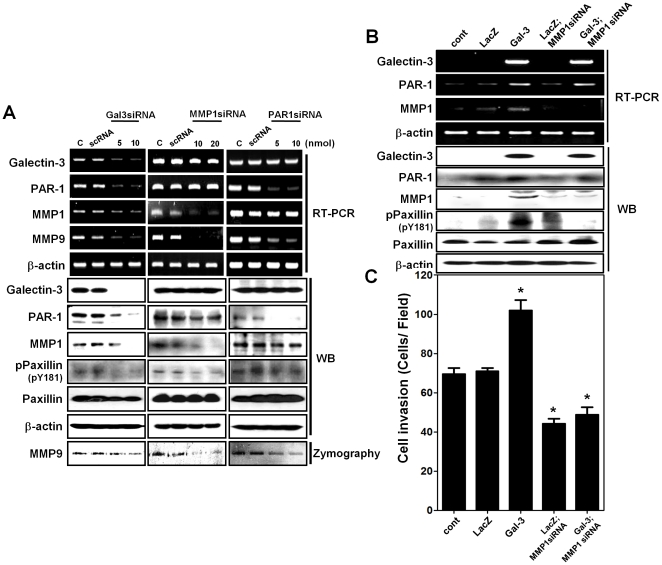
Galectin-3 mediated MMP-1 expression promotes gastric cancer cell invasion through PAR-1 activation. **A,** galectin-3, PAR-1, MMP-1 and MMP-9 mRNA and galectin-3, PAR-1, MMP-1, phospho-Paxillin (pY181) and paxillin protein levels after transfection with scRNA or each siRNAs of galectin-3, PAR-1, MMP-1 in MKN-28 cells. Total RNA and protein obtained after transfection for 48 hr and cells were harvested and analyzed by RT-PCR and western blot. MMP-9 activity was assayed by gelatin zymography using the medium of MKN-28 cells. **B,** mRNA expression levels of galectin-3, PAR-1 and MMP-1 by RT-PCR; protein expression levels of galectin-3, PAR-1, MMP-1, phospho-paxillin (pY181) and paxillin by western blot analysis in SNU-638 cells, which were infected with lenti-virus containing LacZ or galectin-3, and then transfected with MMP-1 siRNA or scRNA for a negative control. **C,** Invasion assay of SNU-638 cells infected with lenti-virus containing LacZ or galectin-3 cells, and then transfected with MMP-1 siRNA or scRNA for a negative control. The data are presented as histogram (* *p*<0.001 vs. Cont group).

MMP-1 has been reported to regulate PAR-1 activation through cleavage of PAR-1 tethered intracellular signaling, and to influence cell invasion [21]. As presented in Figure S1, we showed that galectin-3 regulated MMP-1 expression. Therefore, we confirmed the interrelationships of galectin-3, MMP-1 and PAR-1. We analyzed the mRNA expression levels of MMP-9, which is a downstream target of PAR-1 [8], after silencing galectin-3, MMP-1 and PAR-1 individually (Figure 3A). While galectin-3 silencing reduced the expression of all three molecules (MMP-1, PAR-1 and MMP-9), MMP-1 silencing reduced only MMP-9 expression and not those of galectin-3 or PAR-1. Interestingly, PAR-1 silencing produced the same effects as MMP-1 silencing. We also detected the phosphorylation of the cytoskeletal protein paxillin (pY181), a hallmark of PAR-1 activation [22], [23]. After silencing galectin-3, MMP-1 and PAR-1 individually, the phosphorylaion of paxillin was decreased, which suggested galectin-3 also regulated PAR-1 activity. We also checked the reduction of MMP-9 activity after silencing of galectin-3, MMP-1 and PAR-1 individually (Figure 3A).

Galectin-3 over-expressing SNU638 cells showed increased expression levels of MMP-1 and PAR-1 protein as well as their mRNAs, in addition to phosphorylated paxillin (pY181). In these cells, MMP-1 silencing reduced the phosphorylation of paxillin without changing the expression of galectin-3 or PAR-1 (Figure 3B). Moreover, MMP-1 silencing reduced cell invasion, which is triggered by galectin-3 over-expression (*p*<0.001) (Figure 3C), which implies that galectin-3 increased MMP-1 expression leading to activation of PAR-1 signaling.

### Over-expression of MMP-1 in cancer cells increases cell invasion through the activation of PAR-1 signaling

**Figure 4:**
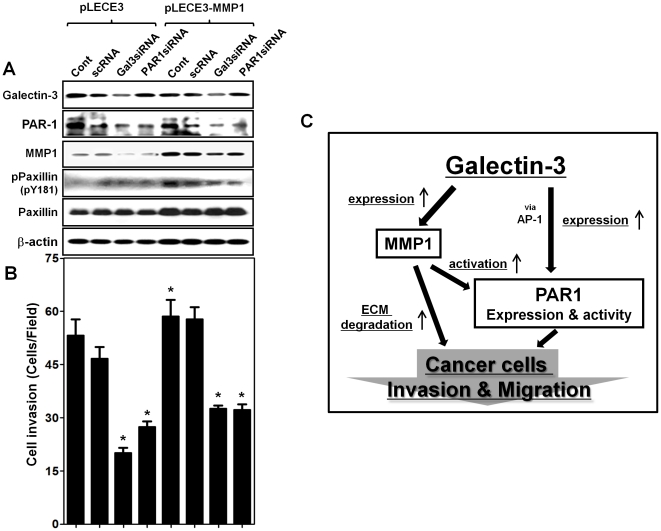
Over-expressed MMP-1 influences gastric cancer cell invasion and schematic model of galectin-3 regulate gastric cancer cell invasion and migration. **A,** protein expression of galectin-3, PAR-1, MMP-1, phospho-Paxillin (pY181) and paxillin levels were measured by western blot analysis, after infection with lenti-viral construct containing pLECE3 (vector only) and pLECE3-MMP-1 in AGS cells, transfected with galectin-3 or PAR-1 siRNAs. **B,** Cell invasion performed by the above cells present as a histogram (* *p*<0.001 vs. Cont group). **C,** Galecitn-3 enhances PAR-1 expression via binding with AP-1 transcription factor, also, galectin-3 regulation of MMP-1 expression. MMP-1 increase causes dual effects in gastric cancer invasion; 1) cleavage of PAR-1 tethered ligand and PAR-1 activation 2) degradation of extracellular matrices (ECM).

We confirmed that up-regulation of MMP-1 by galectin-3 is important for the activation of PAR-1 signaling and increased gastric cancer cells invasion. A lentivirus (pLECE3 Vector) containing MMP-1 expression cassette regulated by CMV promoter was prepared and infected in AGS gastric cancer cells (Figure 4A–B). As expected, MMP-1 over-expression increased the invasion potential of gastric cancer cells, and PAR-1 silencing significantly reversed this increase (*p*<0.001) (Figure 4B). This was also confirmed by the fact that over-expression of MMP-1 increased the phosphorylation of paxillin, and that additional silencing of PAR-1 blocked such phosphorylation (Figure 4A). In MMP-1 overexpressing cells, galectin-3 silencing reduced the expression of PAR-1, but not that of MMP-1, and also the phosphorylation of paxillin and cell invasive potential (Figure 4A–B). These observations suggested that over-expression of MMP-1 increased cancer cell invasivity by activation of PAR-1 signaling. Taken all together, these results suggested that galectin-3 increased the expression of both PAR-1 and MMP-1, and that the increased MMP-1 expression activated PAR-1 signaling, which in turn, facilitated cancer cell invasion. These events are schematically depicted in Figure 4C.

### Silencing of galecin-3, PAR-1 or MMP-1 blocks gastric cancer cell migration *in vivo* in a zebrafish model

**Figure 5:**
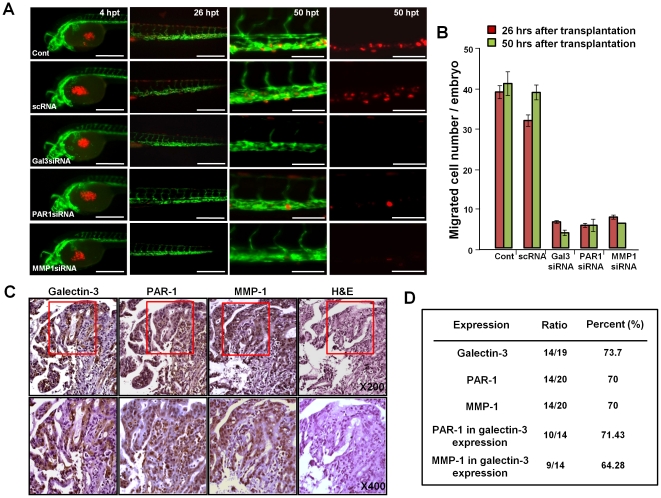
Studies on the migration of gastric cancer cells in the zebrafish model. **A,** At 53 hours post fertilization (hpf), the transplanted AGS cells which transfected galectin-3, PAR-1, MMP-1 siRNA and scRNA (red) were located in the center of yolk sac of living transgenic zebrafish in which embryonic vessels are visualized with green fluorescence at 4 hours post transplantation (hpt); up to 50 hpt were clearly detected only cancer cells. **B,** The number of migrated cells per embryo were counted, and shown as histogram. These data were derived from three replicated experiments. *Scale bars*, 200 µm and 50 µm in last panels. **C** and **D,** Increased expression of galectin-3, PAR-1 and MMP-1 in gastric cancer patients. **C,** Expression and localization of galectin-3, PAR-1 and MMP-1 in malignant tissues from gastric cancer patients using immunohistochemical staing (brown) with H&E by fluorescence microscopy. Magnification: (upper panel)×200; (lower panel)×400. **D,** mRNA of galectin-3, PAR-1 and MMP-1 levels in tissues of gastric cancer patients detected by RT-PCR (Figure S3). Malignant and normal tissues were obtained from 20 gastric patients subjected to RT-PCR, quantified and analyzed by NIH image analyzer. Higher expression levels of galectin-3, PAR-1 and MMP-1 in malignant tissues are shown as percentage increases over their levels in normal tissues.

Transgenic zebrafish, *Tg(kdrl:EGFP)^s843^* [24], express EGFP specifically in their vasculature. The corresponding zebrafish embryos are transparent with green fluorescent vessels. We employed these fish to conduct *in vivo* studies of human gastric cancer cell migration (Figure 5A). When the red fluorescent labeled AGS cells were injected into the yolk sac of the zebrafish embryos at 48 hpf (hours post fertilization), the majority of transplanted AGS cells were located in the center of the yolk sac of the embryos by 4 hrs post transplantation (hpt). After 26 hpt, both the normal and scRNA transfected cells migrated into trunk region, and resided in the vessel of trunk and/or tail of the embryos at 50 hpt. However, the number of migrated AGS cells, in which each of galectin-3, PAR-1 or MMP-1 are silenced, significantly decreased (Figure 5B). We also counted the number of zebrafish embryos that display migrating cells and the results are shown (Figure S2). Eighty four to ninety three percent of zebrafish embryos which were transplanted with control cells or scRNA treated cells displayed cells migrating into their trunk and tail vessels at 50 hpt. However, only 7 to 11% of embryos which were transplanted with cells in which galectin-3, PAR-1 or MMP-1 were silenced, displayed migrating cells. These findings suggested, that first, the zebrafish embryo model can be used to monitor the migration of gastric cancer cells as a living animal, and second, that silencing of each of galectin-3, PAR-1 and MMP-1 caused significant reduction in gastric cancer cell migration *in vivo*.

### Expressions of galectin-3, PAR-1 and MMP-1 are elevated, in parallel, in the malignant tissues of gastric cancer patients

We determined the mRNA and protein expression levels of galectin-3, PAR-1 and MMP-1 in normal and malignant tissues from 20 gastric cancer patients, then employed RT-PCR using an image J analyzer. As shown in Figure 5C and Figure S3, 73.7% of the cancer patients showed higher expression levels of galectin-3 and 70% of them showed higher PAR-1 and MMP-1 levels in their malignant tissues than in their normal counterparts. Moreover, among galectin-3 positive patients, 71.4% were also PAR-1 positive while 64.3% were also MMP-1 positive (Figure 5D). These data indicated that there is a parallel increase in the expression of galectin-3 as well as PAR-1 and MMP-1, in the malignant gastric tissues. We also found that these proteins co-localized in the malignant areas of the tissues, and not in non-malignant areas (Figure 5C). Galectin-3 was present in both the cytosol and nucleus, while PAR-1 and MMP-1 were seen generally in the cytosol and membrane within the same area (Figure 5C). These findings suggested that both PAR-1 and MMP-1 expressions significantly correlated with galctin-3 expression in gastric cancer patients.

## Discussion

This study demonstrated that galectin-3 enhanced gastric cancer cell migration and invasion. Mechanistically, this linked galectin-3 to up-regulation of PAR-1 cell surface receptor, and the activity MMP-1 protease. This is in agreement with reports demonstrating a correlation between expression of PAR-1 and tumor invasion and metastasis in gastric and several other cancers [25], [26], [27]. This study is the first to demonstrate a direct interaction of galectin-3 and AP-1 complex components, c-Jun and Fra-1, and regulation of PAR-1 expression by AP-1 transcriptional factor. Since it has been already shown by others that over-expression of Fra-1 promotes cancer cell invasion and metastasis [28], up-regulation of PAR-1 by c-Jun and Fra-1 seemed to be a possible mechanism explaining the linkage of galectin-3 induced up-regulation of PAR-1 cell surface receptor, and the activity MMP-1 protease. This possibility is supported by our finding of parallel and co-localized expressions of galectin-3 and PAR-1 in malignant tissues of gastric cancer patients.

It is a novel observation that galectin-3 increases MMP-1 expression. Clearly, the over-expression of MMP-1 can increase gastric cancer cell invasion activity by blocking cell to cell and cell to matrix interactions by ECM degradation. Therefore, an increase in MMP-1 expression promoted by galectin-3 might have dual effects, namely, PAR-1 activation and ECM degradation, and both are critical for gastric cancer metastasis. However, how galectin-3 regulates MMP-1 expression is still unclear, because the expression of MMP-1 is regulated by a series of complex events including cross-talk between several transcriptional factors, such as AP-1, signal transducer and activator of transcription-3, MAP kinase, and hypoxia-inducible factor-1 in hypoxia [29], [30], [31].

Establishing the zebrafish (*Danio rerio*) embryo model for studying the migration and invasion of gastric cancer cells is a particular accomplishment of this study because suitable animal models were not available for studying human gastric cancer metastasis until now. The zebrafish model to study genetically engineered cancers and/or tumor xenografts, has many advantages, including feasibility of forward and reverse genetic analyses, transparency of the embryos [32], and suitability for GFP labeling of vessels [32], [33], [34]. This study showed that RFP labeled gastric cancer cells invaded blood vessels while galectin-3, MMP-1 or PAR-1 silenced gastric cancer cells could not. Thus, the zebrafish embryo seems to be a promising model for studying invasion and metastasis of cancer cells, although further studies are clearly needed to confirm this finding.

In conclusion, our studies demonstrated that galectin-3 accelerated gastric cancer cell motility by up-regulating of PAR-1 and MMP-1. Further studies are clearly needed to establish the role of galectin-3 in cancer metastasis and its suitability as a therapeutic target for selected cancers.

## Materials and Methods

### Tissues

Pairs of 2 mm sized biopsy specimens were obtained from gastric adenocarcinoma tissues of 20 patients undergoing diagnostic endoscopy and endoscopic submucosal dissection, at National cancer center in Korea, after obtaining their written informed consents. The tissue samples were frozen in liquid nitrogen at −70°C immediately after biopsy until use. Some of the tissue samples were paraffindized for immunohistochemical studies, as needed. These studies were approved by the Institutional Review Board of National cancer center (# NCCNSH 03-024).

### Cell Culture and siRNA Transfections

Moderately differentiated human gastric adenocarcinoma cell lines, AGS, MKN-28 and SNU-638 were obtained from the Korea Cell Line Bank and maintained as described previously [35]. siRNAs used in transfections, were all provided by Invitrogen (Carlsbad, CA). Their sequences are galectin-3(LGAL3) siRNA; 5′-AUAUGAAGCACUGGUGAGGUCUAUG-3′, PAR-1 siRNA; 5′-CCCAUCUGUGUACACCGGAGUGUUU-3′, and MMP-1 siRNA; 5′-GGAGAAAUAGUGGCCCAGUGGUUGA-3′. Transfections were carried out using Lipofectamine RNAiMAX reagent (Invitrogen), following the manufacturer's instructions. Induction of transient galectin-3 overexpression in SNU-638 cells was achieved using pcDNA3.1/NT-galectin-3 with pcDNA3.1/NT-GFP. For normalization control treatment, the gastric cancer cell lines were treated with 1 µg by LTX reagents (Invitrogen).

### RNA isolation and RT-PCR analysis

Total RNA was isolated from human gastric cancer cells and patient tissue samples, using TRIzol Reagent (Invitrogen) following the manufacturer's instructions. Reverse transcriptase PCR was performed using a Reverse Transcription system (Promega Corp. USA), following the manufacturer's instructions. The primers were used: 5′-ATGGCAGACAATTTTTCGCTCC-3′ (sense) and 5′-ATGTCACCAGAAATTCCCAGTT-3′ (anti-sense) for human LGAL3(galectin-3) gene; 5′-CCAAAACTGAGCATAAGTCC-3′ (sense) and 5′-AGGATGGAGCAAATGTAGTG-3′ (anti-sense) for human F2R (PAR-1) gene; 5′-ACAGCTTCCCAGCGACTCTA-3′ (sense) and 5′-CAGGGTTTCAGCATCTGGTT-3′ (anti-sense) for human MMP-1 gene; 5′-CTCGAACTTTGACAGCGACA-3′ (sense) and 5′-GCCATTCACGTCGTCCTTAT-3′ (anti-sense) for human MMP-9 gene; 5′-AGCCTCGCCTTTGCCGA-3′ (sense) and 5′-CTGGTGCCTGGGGCG-3′ (anti-sense) for human β-actin gene; 5′-GGCTGCTTTTAACTCTGGTA-3′ (sense) and 5′-ACTTGATTTTGGAGGGATCT-3′ (anti-sense) for human glyceraldehyde 3-phosphate dehydrogenase (GAPDH). PCR was performed in a 20 µl volume using an Ex taq (Takara). The amplification cycle (denaturation 95°C for 1 min, annealing 60°C for 1 min and extension step at 72°C for 2 min) was repeated 32 times followed by final extension for 10 min at 72°C.

### Construction of galectin-3 expressing lenti-viral vector and infection

The full-length human galectin-3 expressing construct pcDNA3.1-NT-GFP-gal3, the pcDNA3.1-NT-GFP vector and the lenti-viral vector to over-express LacZ, galectin-3 (1–250) in pLL3.7 have been previously described [2]. Human F2R was cloned into pLECE3 in BamH1 and Not1 restriction enzyme sites, creating the pLECE3-F2R (PAR-1) construct. Full-length cDNAs of F2R, MMP-1, and control mRFP were used for PCR amplification, along with the primers listed in Data S1, and resulting PCR fragments were cloned into the pLECE3 vector using Pac1 and Hpa1 restriction enzyme sites, creating the pLECE3-MMP-1 construct. Also, the mRFP site of the pGEM-T-Easy vector was transfered to the pLL3.7 vector replacing the GFP region using Age1 and EcoR1 restriction enzyme sites, to make pLL3.7-mRFP. Lenti-viral vector production has been previously described [2].

### Western blot analysis

Cell lysate extractions were prepared with RIPA buffer (1% NP-40; 0.1% sodium dodecyl sulfate; 0.5% desoxycholate; 150 mM NaCl; 50 mM Tris, pH 7.5) and protease inhibiter cocktail. 20 µg total protein of each lysate was resolved in 8–12% SDS PAGE gels and electro-transferred to PVDF membrane, and then blocked in 5% skim milk in 0.05% Tween-20 with 1×PBS (PBST). Polyclonal primary antibodies, anti-Galectin-3, anti-ThrombinR (PAR-1), anti-c-Jun, anti-Fra-1 and anti-β-actin (Santa Cruz), anti-MMP1 (Calbiochem), anti-Paxillin and anti-phospho paxillin (pY181) (Epitomics) were incubated with blots at 1∶1000 dilution in minimal volumes of 5% BSA (Bovine serum albumin) in PBST buffer for 1 hr room temperature or over-night in 4°C. Anti-mouse or anti-rabbit goat-HRP-conjugated secondary antibodies (GE healthcare UK limited) were incubated at 1∶5000 dilution of 5% BSA in PBST buffer for 1.5 hr at room temperature. Proteins of interest were detected by enhanced chemiluminescence (ECL) (Amersham Corp, Arlington Heights, IL, USA).

### Immunoprecipitation

Cell lysate extracts were prepared with IP+ buffer (20 mM Hepes, 1% Triton X-100, 150 mM NaCl, 1 mM EDTA, 1 mM EGTA, 100 mM NaF, 10 mM sodium pyrophosphate, 1 mM sodium orthovanadate, 0.2 mM PMSF, 10 µg/ml protease inhibitor cocktail) in ice for 30 min. After debris removal by centrifugation (13200 rpm at 4°C for 20 min), the supernatants were subjected to a preclearing step with protein A/G Agarose beads at 4°C for 30 min in rotator. Supernatants obtained after a brief centrifugation (2000 rpm at 4°C for 4 min) were subjected to immunoprecipitation using an anti-galectin3 and normal mouse IgG (negative control). Immunoprecipitates were washed twice in IP- buffer (20 mM Hepes, 1% Triton X-100, 150 mM NaCl, 1 mM EDTA, 1 mM EGTA, 100 mM NaF, 10 mM sodium pyrophosphate). After 30 µl 2×SDS-sample buffer addition, followed boiling at 95°C for 5 min. After supernatants obtained after a brief centrifugation, their protein expression levels were determined through the western blot analysis performed.

### Immunohistochemistry

For galectin-3 and PAR-1 immunohistochemistry, deparaffinized gastric cancer patients tissue sections were left in a microwave for 15 min in citrate buffer (Vector laboratories), and then incubated with 3% H_2_O_2_ for 15 min to block endogenous peroxidase, followed by incubation with 10% normal goat serum (Vector laboratories) in 1×PBS for 10 min. Primary antibodies were used a anti-galectin-3 (1∶200) anti-MMP1 (Epitomics) and anti-PAR-1 (1∶200) antibodies (Santa cruz), and next step was done according to instructions of Vectastain®ABC kit (Vector laboratories). The products were visualized by diaminobenzidine (DAB) kits (Vector laboratories).

### Gelatin zymography

Conditioned media were obtained from cultures of MKN-28 gastric cancer cell lines and concentrated using vivaspin 6 (Sartorius). The concentrated supernatants in 6× Foz buffer were separated on 10% SDS gels containing 1 mg/ml of gelatin. Each was incubated in 100 ml of zymography reaction buffer and stained with Bio-safe™ coomassie G-250 (Bio-Rad Laboratories). After destaining, sharp transparent bands indicating gelatinolytic activity were visualized on a blue background.

### Transfilter migration and invasion assays

For the transfilter migration and invasion assay, we used MKN-28 and SNU-638 cells. The cells were transfected with siRNAs for 1 day, isolated and added to upper chambers of pore inserts Transwell with collagen type l (BD bioscience) coated filters in migration assay, and matrigel (BD bioscience) coated filters, for invasion assays. RPMI 1640 with 10% FBS and 1% antibiotics (Gibco) was added to the lower chamber followed by incubation for 20 hrs. Migrating and invading cells were quantified after H&E staining. Each experiment was carried out in triplicate and data presented as mean values.

### Chromatin immunoprecipitation assays

Chromatin immunoprecipitation (ChIP) assays were performed using a ChIP assay kit (Millipore). Samples were applied on dishes after galectin-3 siRNA treatment and assays were conducted following the manufacturer's instructions. Anti-Galectin-3, anti-c-Jun, anti-Fra-1 and normal mouse/rabbit IgG were used to immunoprecipitate DNA containing complexes. Prior to PCR, primers were prepared for AP-1 with PAR-1 promoter binding sites −666 (5′-CACTGT CGACGTCTCCACAT-3′) and PAR-1 promoter site −307 (5′-GGGCCTAGAAGTCCAA ATGA-3′). PCR was performed with an Ex taq (Takara).

### Luciferase assays

An AP-1 luciferase reporter plasmid was obtained from Dr. Sung-Pil Yoon of the National cancer center in Korea and studies of the effects of galectin-3 on AP-1 transcriptional activity in lacZ and galectin-3 over-expressing cells were performed in accordance with a previous report [2]. After 48 hours, the cells were harvested and luciferase activity was measured using a luciferase assay system (Promega) according to the manufacturer's instructions. Each experiment was carried out in triplicate and data presented as mean values.

### Cell migration assay using the zebrafish model

The transgenic zebrafish line *Tg(kdrl:EGFP)^s843^* [24], in which EGFP is specifically expressed in the vasculature, was maintained in accordance with accepted standard operating procedures [36] approved by the Institutional Animal Care and Use Committee at National Cancer Center in Korea; permit number: NCC 11-116. We obtained zebra-fish embryos from national cancer center zebrafish aqua-room. Cancer cell transplantation was performed according to Nicoli et al [37] with slight modifications. Briefly, dechorionated embryos were anesthetized with tricaine (Sigma) and cancer cells were injected (150 to 200 per embryo) into the centers of embryonic yolk sacs at 48 hpf (hours post fertilization) using a Micromanipulator (Narishige) and a Pneumatic Picopump (WPI) equipped with a borosilicate glass needle. Embryos were maintained after transplantation procedures and were examined for migrating cancer cells by fluorescence microscopy. For imaging, embryos and larvae were treated with N-phenylthiourea (Sigma) from 12 hpf to prevent pigmentation and examined with a fluorescent inverted microscope (Carl Zeiss) under epifluorescence optics. Fluorescent images were captured with a CCD camera (Carl Zeiss). Stable fluorescent gastric cell lines AGS (mRFP) were generated by lenti-virus infection with pLL3.7-mRFP followed by FACS sorting.

### Microarray data analysis

Microarray was performed in AGS, gastric cancer cell line after silencing of galectin-3 as described previously [6]. Microarray results were uploaded on GEO (GSE29630).

### Statistics analysis

Data were presented as mean ± SD from at least 5–10 images obtained from three independent experiments using microscope (×100, ×200), unless otherwise indicated. Statistical analysis was performed by one-way ANOVA test using Prism software. Data was considered significant if *p*<0.05.

## Supporting Information

Figure S1 **Silenced galectin-3 with siRNA in gastric cancer cells resulted in changes in cell motility related gene expression.** **A,** Among DNA microarray analysis, we chose cell motility related gene MMP-1, MMP-3, FSCN1(Fascin-1), F2R(PAR-1), TIMP-2, SERPINE1(PAI-1) and showed fold change ratio. **B,** Detection of mRNA expression of MMP-1, MMP-3, FSCN1(Fascin-1), F2R(PAR-1), TIMP-2, SERPINE1(PAI-1) using by PCR. β-actin was used as a normalization control.
(TIF)

Figure S2 **The number of zebrafish embryos with migrated cells ratio.** Detection of gastric cancer cell AGS (RFP) numbers after transplanted of AGS (RFP) in zebrafish fish embryo with treatment specific siRNA (galectin-3, MMP-1 and PAR-1) with negative control scRNA (Figure 5A–B). It showed number of migrated cell per embyos was counted and shown as percentage.
(TIF)

Figure S3 **RT-PCR analysis of mRNA level of galectin-3, MMP-1 and PAR-1 in gastric cancer patients.** mRNA expression of galectin-3, PAR-1 and MMP -1 in gastric cancer patient tissues were detected by RT-PCR experiments. GAPDH was used as a normalization control.
(TIF)

Data S1 **Primer list of of F2R, MMP-1, and control mRFP were used for PCR amplification.**
(TIF)
