# Supplementary figures and images for: Galectin-3 Facilitates Cell Motility in Gastric Cancer by Up-Regulating Protease-Activated Receptor-1(PAR-1) and Matrix Metalloproteinase-1(MMP-1)
Source: PLoS One. 2011 Sep 22;6(9):e25103. doi: 10.1371/journal.pone.0025103 (PMC3178590; doi:10.1371/journal.pone.0025103)

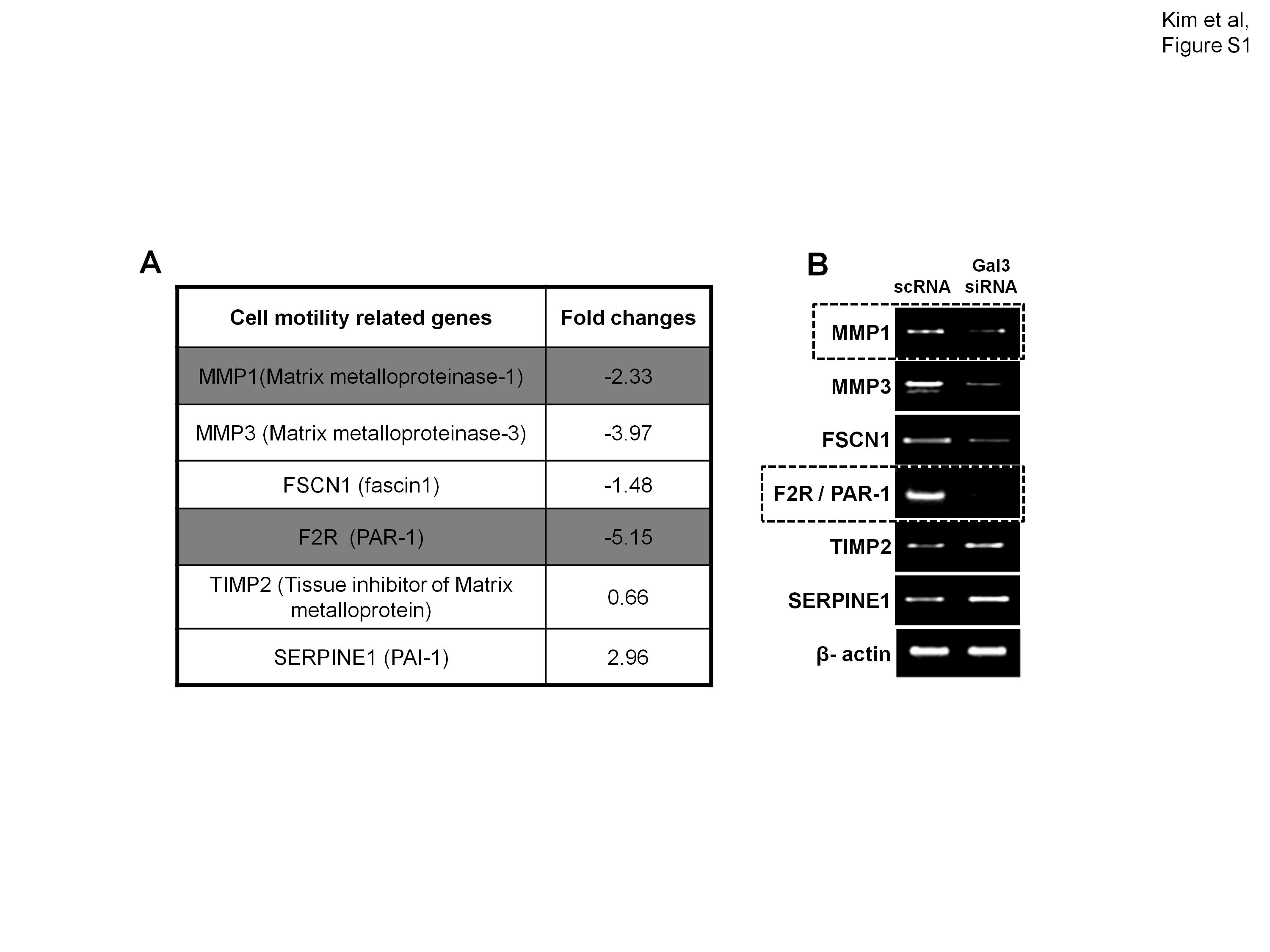

Supplement: Figure S1 — Silenced galectin-3 with siRNA in gastric cancer cells resulted in changes in cell motility related gene expression. A, Among DNA microarray analysis, we chose cell motility related gene MMP-1, MMP-3, FSCN1(Fascin-1), F2R(PAR-1), TIMP-2, SERPINE1(PAI-1) and showed fold change ratio. B, Detection of mRNA expression of MMP-1, MMP-3, FSCN1(Fascin-1), F2R(PAR-1), TIMP-2, SERPINE1(PAI-1) using by PCR. β-actin was used as a normalization control. (TIF) [file pone.0025103.s001.tif]

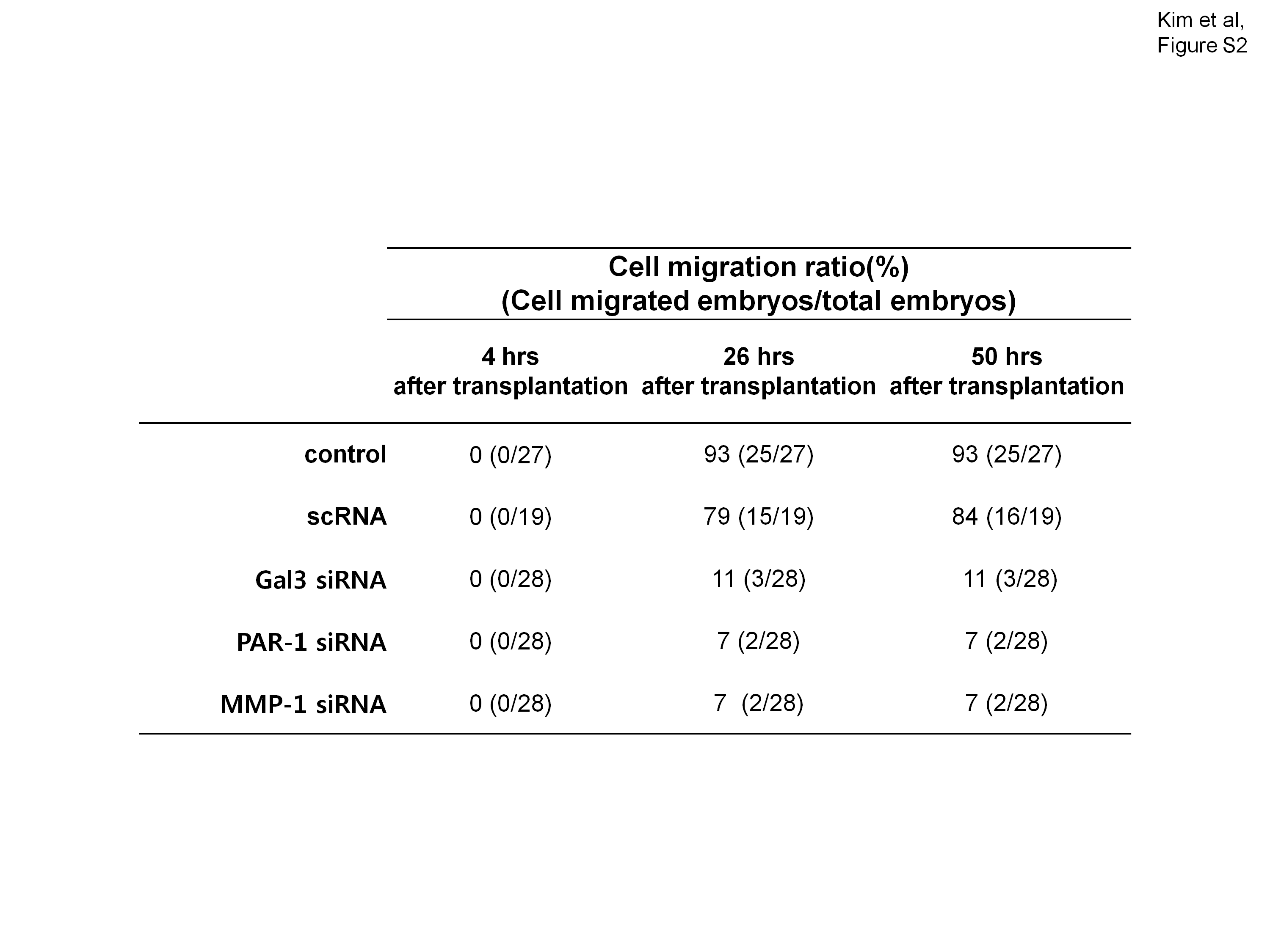

Supplement: Figure S2 — The number of zebrafish embryos with migrated cells ratio. Detection of gastric cancer cell AGS (RFP) numbers after transplanted of AGS (RFP) in zebrafish fish embryo with treatment specific siRNA (galectin-3, MMP-1 and PAR-1) with negative control scRNA (Figure 5A–B). It showed number of migrated cell per embyos was counted and shown as percentage. (TIF) [file pone.0025103.s002.tif]

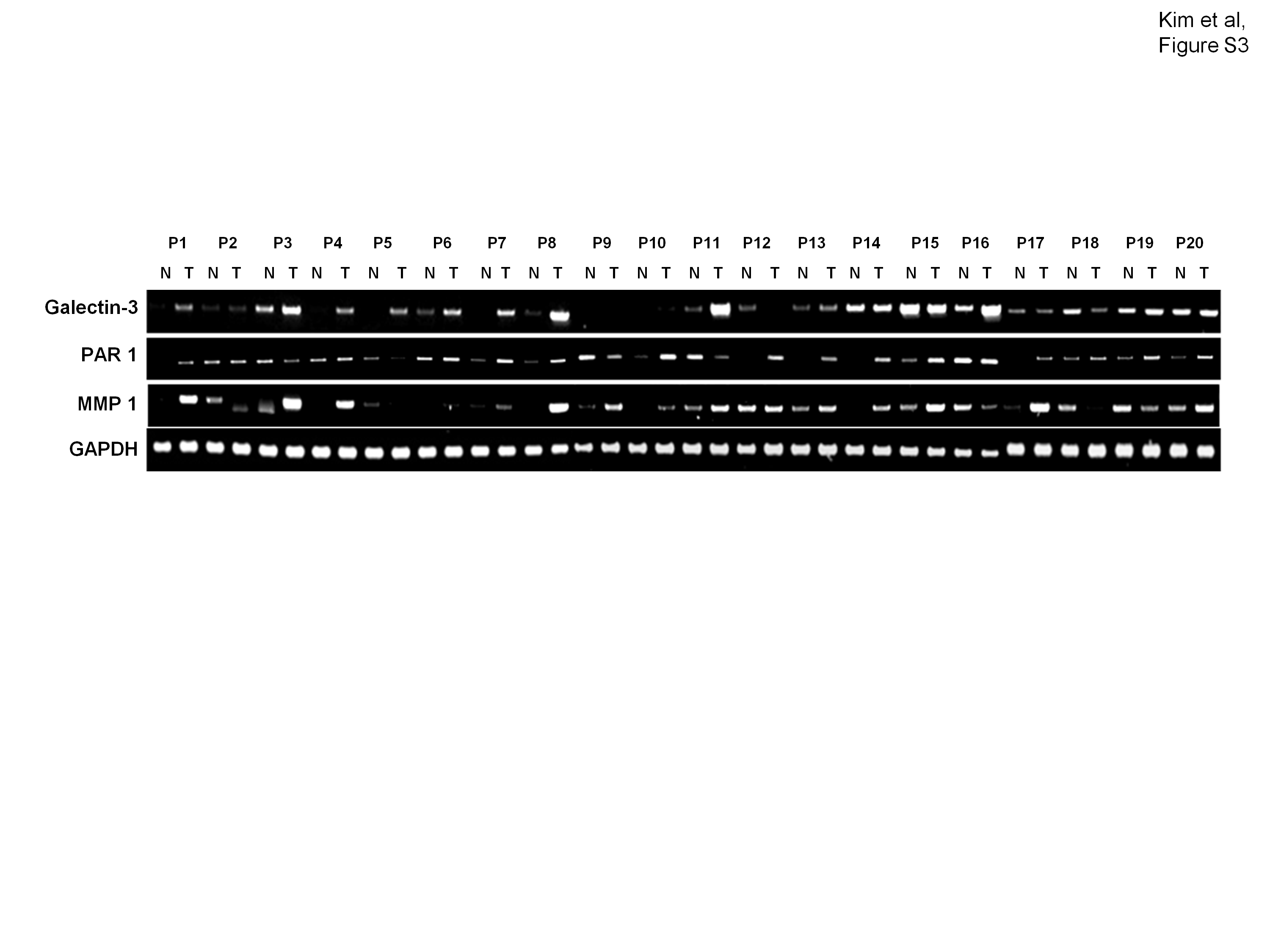

Supplement: Figure S3 — RT-PCR analysis of mRNA level of galectin-3, MMP-1 and PAR-1 in gastric cancer patients. mRNA expression of galectin-3, PAR-1 and MMP -1 in gastric cancer patient tissues were detected by RT-PCR experiments. GAPDH was used as a normalization control. (TIF) [file pone.0025103.s003.tif]

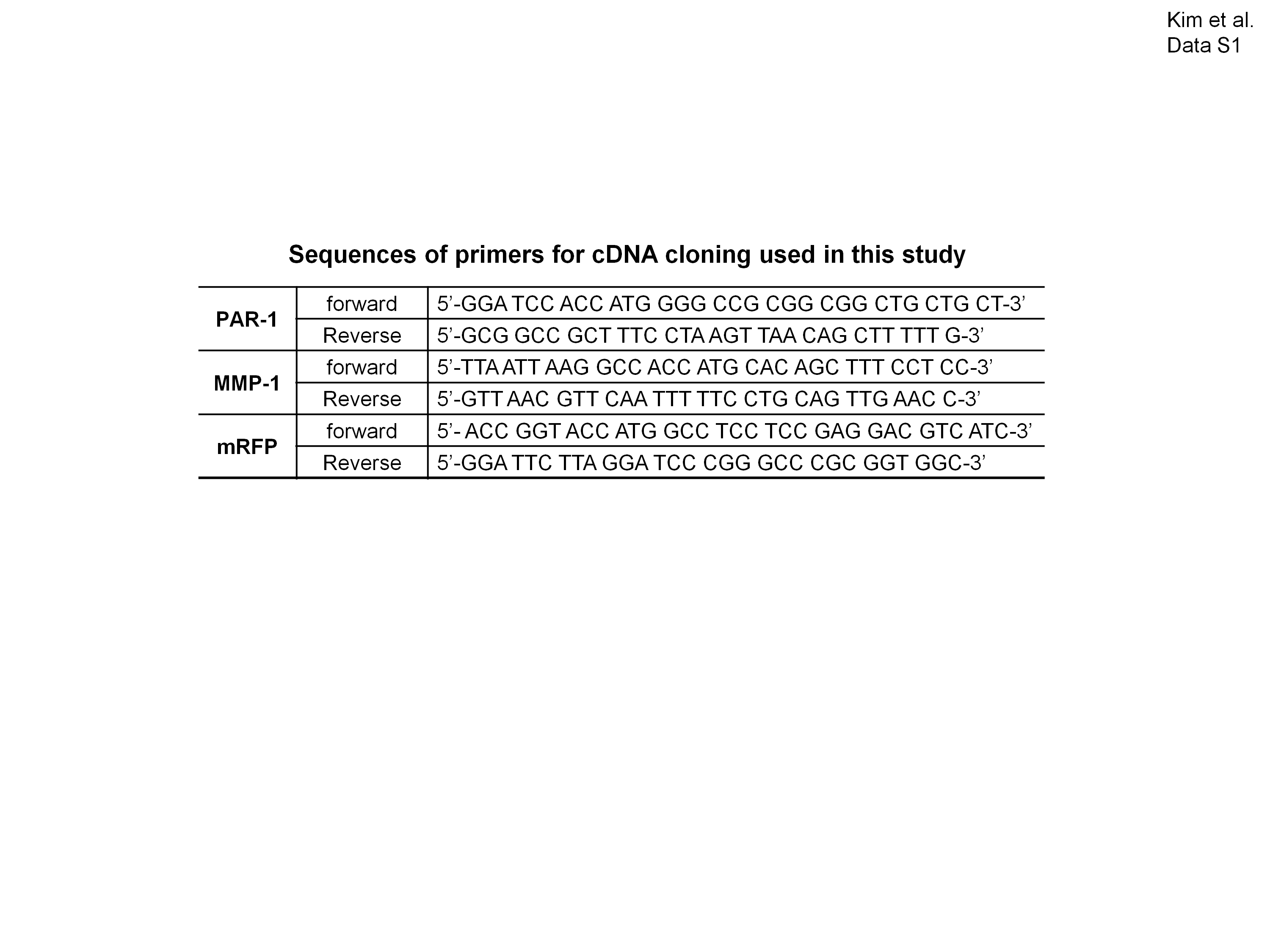

Supplement: Data S1 — Primer list of of F2R, MMP-1, and control mRFP were used for PCR amplification. (TIF) [file pone.0025103.s004.tif]
